# Haloalkane induced hepatic insult in murine model: amelioration by Oleander through antioxidant and anti-inflammatory activities, an in vitro and in vivo study

**DOI:** 10.1186/s12906-016-1260-4

**Published:** 2016-08-11

**Authors:** Priyankar Dey, Somit Dutta, Anashuya Biswas-Raha, Mousumi Poddar Sarkar, Tapas Kumar Chaudhuri

**Affiliations:** 1 Cellular Immunology Laboratory, Department of Zoology, Life Science Building, University of North Bengal, PO: Raja Rammohunpur, Siliguri, 734013 West Bengal India; 2 Chemical Signal and Lipidomics Laboratory, Department of Botany, University of Calcutta, Kolkata, 700019 India

**Keywords:** Hepatoprotective, Inflammation, Liver, Nerium, Oxidative stress, Xenobiotic

## Abstract

**Background:**

*Nerium oleander* L. (syn. *Nerium indicum* Mill, *Nerium odorum* Aiton) belongs to the family Apocynaceae. It is used for its anti-inflammatory, anti-diabetic, anti-cancer and hepatoprotective activities in traditional medicine. Previous pharmacognostic studies suggested that 70 % hydro-methanolic extracts of oleander possess potent free radical scavenging and anti-inflammatory activities, both of which are helpful against hepatotoxicity.

**Methods:**

Hydro-methanolic extracts of oleander stem and root were evaluated for their hepatoprotective activities in acute CCl_4_ intoxicated mouse through in vitro and in vivo studies. Silymarin was used as positive reference. Antioxidant enzymes, pro-inflammatory markers and liver enzymatic and biochemical parameters were studied. The extracts were further chemically characterized using Fourier Transform Infrared (FTIR) spectroscopy and Gas chromatography-mass spectrometry (GC-MS).

**Results:**

CCl_4_ toxicity caused fatty liver formation by increase of relative liver weight (32.53 g) compared to control group (16.08 g). The elevated liver enzymatic and biochemical parameters due to CCl_4_ toxicity were considerably normalized by the extracts treatment under both in vivo and in vitro models. Oleander stem (NOSE) and root (NORE) extracts increased the reduced hepatic catalase activity 27.37 and 25.25 %, whereas peroxidase activity was increased 18.19 and 22.78 %, respectively. The extent of lipid peroxidation was significantly (*p* < 0.01) lowered 20.76 % (NOSE) and 21.12 % (NORE) compared to CCl_4_ group. The levels of pro-inflammatory tumor necrosis factor-α (TNF-α) was lowered 71.33 % (NOSE) and 61.60 % (NORE). Histopathological study demonstrated substantial reduction of hepatocellular necrosis, fatty infiltration, sinusoidal dilation, bile duct proliferation, vascular congestion, leukocyte infiltration in the silymarin and extract treated groups. Furthermore, various bioactive compounds were identified in the extracts such as apocynin, tocopherol, squalene, vanillin, isoeugenol, amyrin, lupeol etc.

**Conclusion:**

The present study provided convincing evidence that oleander extracts possess potent hepatoprotective capacity which was primarily governed by its antioxidant and anti-inflammatory activities. The collegial bioactivities of the phytochemicals may be accredited behind the hepatoprotective activity of oleander.

**Electronic supplementary material:**

The online version of this article (doi:10.1186/s12906-016-1260-4) contains supplementary material, which is available to authorized users.

## Background

The liver is the largest organ of our body, contributing about 2 % of the total body weight. The liver is associated with most of the metabolic and physiological process including growth, immunity, nutrition, energy metabolism and reproduction [1]. Production of different coagulation factors, antithrombin, thrombopoietin, angiotensinogen, insulin-like growth factor 1, albumin, catabolism of bilirubin and various hormones takes place in the hepatic system. Liver performs central role in the biotransformation and metabolism of xenobiotic compounds, which in some case results in liver damage [2]. Though the liver possesses tremendous regenerative capacity, but very often subclinical live injury occurs due to toxic chemicals and their metabolic intermediates. Around the globe, drug induced hepatotoxicity has emerged as a serious medical concern where 10 % cases of acute liver failures are associated with idiosyncratic hepatotoxicity [3]. Drug induced hepatotoxicity has now appeared as the leading cause behind acute liver failure among the US patients [4]. The liver is one of the decisive organs of the body and therefore, requires safeguard from harmful chemicals.

Persistent exposure to xenobiotics, especially synthetic drugs and environmental chemicals cause serious liver damage due to its portal location in the circulation and central role in detoxification [5, 6]. Most xenobiotics are lipophilic in nature and thus, are poorly excreted by the kidney. The liver performs a critical role in biotransformation of such xenobiotics to more polar form in order to facilitate their excretion [7]. Halogenated alkanes are organic xenobiotics which holds the potentiality to cause severe liver injury [2, 6]. CCl_4_ is a model haloalkane which is extensively used to study the pathophysiological implications of xenobiotic metabolism and hepatoprotective potentialities of natural compounds [8]. Xenobiotic mediated liver damage like CCl_4_ toxicity, is primarily governed by free-radical mediated tissue injury and inflammatory damages. CCl_4_ cause hepatocellular ionic imbalance, oxidative stress, translational inhibition, Ca^2+^ shift, mitochondrial respiratory chain blockage and impairment of β-oxidation [9, 10]. Histopathological signs of CCl_4_ toxicity includes zonal haemorrhagic necrosis, fatty infiltration, sinusoidal dilation, calcification, vascular congestion, bile duct proliferation and leukocyte infiltration [8].

Previously, we have demonstrated that the 70 % hydro-methanolic extracts of oleander possess potent antioxidant capacity [11]. The extracts also demonstrated free radical scavenging activities against various reactive species associated with hepatotoxicity such as hydroxyl (OH^●^), superoxide (O_2_^●−^), peroxynitrite (ONOO^−^), singlet oxygen (^1^O_2_), hydrogen peroxide (H_2_O_2_) and nitric oxide (NO), in addition to potent iron (Fe^2+^) chelation activities, overload of which may lead to haemochromatic liver [12]. It is interesting to note that, a recent in vivo preliminary study demonstrated that *N. indicum* leaf extract ameliorates haemochromatic conditions in mouse [13]. Moreover, another recent study showed that *N. indicum* leaf extract attenuates CCl_4_ mediated hepatotoxicity in Swiss albino mouse [14]. Simultaneously, the same 70 % hydro-methanolic extract of oleander leaf possess anti-inflammatory activities by modulation of Th1/Th2 cytokine balance, inhibition of cyclooxygenase, prostaglandin E_2_ (PGE_2_) and nitric oxide levels in mitogen induced splenic lymphocytes [15, 16]. Moreover, diverse pharmacognostic activities such as anti-diabetic, anti-microbial, antinociceptive, neuroprotective, anti-ulcer, anti-cancer etc. of oleander extracts and oleander derived bioactive components has also been demonstrated previously [17].

Oleander is extensively used as herbal medicine in different parts of the world [17]. The pharmacognostic studies are mostly limited to the bioactivities of its leaf extracts. However, traditional therapies suggests extensive use of oleander stem and root to cure diverse ailments [17]. Therefore, the present study was initiated to evaluate the proposed hepatoprotective potentiality of steam and root extracts of oleander against haloalkane induced hepatic injury in murine model. The study was supported by both in vitro and in vivo experiments. Further, phytochemical characterization were carried out using FTIR and GC-MS methods to reveal the bioactive constituents in the extracts and to correlate the individual bioactivities of the phytochemicals with the proposed hepatoprotective potentiality of oleander stem and root.

## Methods

### Chemicals

All the chemicals were procured from Sisco Research Laboratories Pvt. Ltd. (Mumbai, India) unless otherwise indicated. Silymarin was obtained from Sigma-Aldrich (USA). Fetal bovine serum (FBS), RPMI-1640, antibiotics and EZcount™ MTT (3-(4,5-dimethylthiazol-2-yl)-2,5-diphenyltetrazolium bromide) Cell Assay Kit were purchased from HiMedia Laboratories Pvt. Ltd. (Mumbai, India). Albumin, γ-glutamyl transferase (GGT), lactate dehydrogenase (LDH), alkaline phosphatase (ALP), bilirubin, protein, aspartate transaminase (AST), acid phosphatase (ACP), alanine transaminase (ALT), glucose, urea and cholesterol estimation kits were obtained from Crest Biosystems (Goa, India). TNF-α ELISA kit was procured from Ray Bio (Georgia, United States) and Thiobarbituric acid reactive substances (TBARS) assay kit was purchased from Cayman chemical company (USA). Milli-Q ultrapure water from the departmental central facility was used in the experiments.

### Preparation of plant extract

Stem and root samples of white flowered variety of oleander were collected from the garden of University of North Bengal (26.71°N, 88.35°S), India. The plant materials were identified by senior plant taxonomist Prof. Abhaya Prasad Das of Department of Botany, University of North Bengal. The voucher specimen was stored at the herbarium of Department of Botany, University of North Bengal with an accession number of 09618.

The stem and root samples were washed properly with double distilled water to remove any dust and foreign materials. The samples were then chopped to 0.5 cm pieces and shade dried at laboratory temperature (25 °C). After 20 days, 70 % hydro-methanolic extract of Oleander stem and root were prepared according to the previous method [11]. A schematic representation of the extract preparation method is provided in the Additional file 1. The lyophilized extracts were stored at −20 °C until further use. The final yield of oleander stem (NOSE) and root (NORE) extracts were 11.82 and 15.22 % of dry weight (DW).

### Animal maintenance

Swiss albino mice (6–8 weeks, 20–25 g) of both sex (3 male and 3 female per group) were maintained inside cage bins (Tarson, India) with rice husk bedding in the animal house of the Department of Zoology, University of North Bengal at a constant 12 h photoperiod (temperature 25 ± 2 °C; humidity 55 ± 5 %) with food and water *ad libitum*. All the experiments were approved by the ethical committee, University of North Bengal (No. 840/ac/04/CPCSEA) and conducted in accordance with the legislation for the protection of animals used for scientific purposes.

### Acute toxicity study

OECD guidelines (test 423: Acute oral toxicity – Acute toxic class method, 2002) were followed to study the acute toxicity of the extracts [18]. Mice were divided into eight groups (*n* = 6) and fasted overnight prior to the experiment. NOSE and NORE were administered orally at 250, 500, 1000 and 2000 mg/kg body weight (bw) dose. Each groups were carefully observed at 30 min and then 2, 4, 8, 24 and 48 h for development of any clinical or toxicological symptoms such as tremors, convulsions, salivation, diarrhoea, lethargy, sleep, coma and alteration in respiratory patterns, skin colour, behaviour pattern etc.

### Experimental design: in vivo

Animals were divided into 7 separate groups (*n* = 6) and following treatments were done once each day for 10 consecutive days**:** Control group received normal saline; CCl_4_ group received 1:1 (v/v) CCl_4_ in olive oil; Silymarin group received 1:1 (v/v) CCl_4_ in olive oil and 100 mg/kg bw silymarin; NOSE low group received 1:1 (v/v) CCl_4_ in olive oil and 50 mg/kg bw NOSE; NOSE high group received 1:1 (v/v) CCl_4_ in olive oil and 200 mg/kg bw NOSE; NORE low group received 1:1 (v/v) CCl_4_ in olive oil and 50 mg/kg bw NORE; NOSE high group received 1:1 (v/v) CCl_4_ in olive oil and 200 mg/kg bw NORE.

On 11^th^ day i.e., 24 h after the last treatment, under anaesthesia (2 % ether), blood was collected from the treated animals by cardiac puncture and finally the animals were sacrificed. Blood was allowed to clot for 60 min at room temperature (25 °C) and then serum was collected by centrifuging at 1000 rpm for 5 min. The straw coloured serum was used to study liver marker enzymes. Liver samples were collected and washed with double distilled water to remove blood and used for antioxidant enzymatic assays. Liver tissue required for histological study were collected in Bouin’s solution.

### Liver function test: in vivo

Serum samples from each group were used to study ACP, albumin, globulin, glucose, ALP, bilirubin, cholesterol, LDH, GGT, AST, ALT, total protein and urea levels using commercially available kits (Crest Biosystems).

### Estimation of peroxidase and catalase activities

Peroxidase activity in the liver samples were estimated by measuring the oxidation of guiacol according to a standard method [19]. Catalase activity was measured by degradation of substrate H_2_O_2_ by catalase in the liver tissue samples following the standard method described by Luck [20].

### Experimental design: in vitro

The in vitro hepatoprotective capacity of NOSE and NORE were studied according to previously standardized methods with minor modifications [14, 21, 22]. Briefly, seven groups of primary explant culture of mice hepatocytes were prepared in RPMI-1640 medium (containing 50 U/ml penicillin, 50 U/ml streptomycin and 50 U/ml nystatin) supplemented with 10 % FBS. After 48 h of the culture, the following treatments were done**:** Control had no separate treatment; CCl_4_ group received 25 μl/ml CCl_4_; Silymarin group received 25 μl/ml CCl_4_ and 100 μg/ml silymarin; NOSE low group received 25 μl/ml CCl_4_ and 25 μg/ml NOSE; NOSE high group received 25 μl/ml CCl_4_ and 100 μg/ml NOSE; NORE low group received 25 μl/ml CCl_4_ and 25 μg/ml NORE and NORE high group received 25 μl/ml CCl_4_ and 100 μg/ml NORE.

The plates were incubated for 2 h and then culture supernatants were collected by centrifugation (5000 rpm for 10 min).

### Liver function test: in vitro

Culture supernatants from each group were analysed for ACP, ALP, bilirubin, LDH, AST, ALT and total protein levels using commercially available kits (Crest Biosystems).

### Measurement of lipid peroxidation

The extent of lipid peroxidation was measured in six sets using TBARS assay kit (Cayman) according to the manufacturer’s instructions. In brief, 100 μl serum samples of different groups were mixed with 100 μl sodium dodecyl sulphate (SDS) solution. The tubes were placed on boiling water bath after addition of 4 ml colour reagent. After 60 min incubation, the tubes were kept on ice for 10 min to stop the reaction. Then the solutions were centrifuged (1600 *g*) for 10 min at 4 °C and the absorbance of the supernatants were recorded at 340 nm.

### Measurement of TNF-α

The amount of TNF-α released in culture supernatants were measured using TNF-α ELISA kit (Ray Bio) according to the manufacturer’s instructions. Absorbance was immediately measured at 450 nm using Bio-Rad iMark™ microplate absorbance reader. Standard was run in parallel to the samples.

### Measurement of inhibition of NO

Culture supernatants were used to determine the NO level using the Griess reagent method [23] with some modifications. Briefly, culture supernatants (60 μl) from each group was mixed with 240 μl of Griess reagent (1 % sulfanilamide and 0.1 % N-(1-naphthyl) ethylenediamine hydrochloride in 2.5 % H_3_PO_4_) in a 96-well plate and incubated for 20 min at room temperature. The purple azo-dye formed, was detected at 540 nm.

### Measurement of cell viability

Hepatocyte necrosis results due to CCl_4_ toxicity. Therefore, MTT assay was performed to measure the protection rendered by NOSE and NORE against CCl_4_ mediated toxicity. Hepatocytes were cultured as described under the in vitro experimental section. The cell viability assay was performed in six sets using EZcount™ MTT Cell Assay Kit (HiMedia) according to the manufacturer’s instructions.

### Histopathological studies

Liver samples were removed from the animals of the in vivo experiments after collection of blood and were fixed overnight in 10 % buffered formalin. The samples were subjected to dehydration and then embedded in paraffin blocks. Thick sections (4 μm) of the paraffin embedded livers were cut in a microtome and then dewaxed in xylene, rehydrated in a series of different grades of alcohol and then washed with distilled water for 5 min. Subsequently, the sections were stained with haematoxylin for 40 s and counterstained with eosin for 20 s. The sections were dehydrated in graded alcohol series and washed in xylene. The slides were observed (100× and 400×) for signs hepatic injury using Nikon ECLIPS E200 microscope.

### Fourier Transform Infrared (FTIR) spectroscopy analysis

FTIR spectrophotometry was used to identify the characteristic functional groups in NOSE and NORE. Small quantity (<10 mg) of dried extracts were taken in CaF_2_ vessel and placed in the sample cup of a diffuse reflectance accessory. The IR spectrum was obtained using Shimadzu 8300 FTIR spectrophotometer at ambient temperature. Background correction was made by taking IR spectrum of de-ionized water as the reference in identical condition. The sample was scanned from 400 to 4000 cm^−1^ for 16 times to increase the signal to noise ratio.

### GC-MS analysis

NOSE and NORE were initially bi-fractionated by dissolving in dichloromethane and n-hexane separately. The mixtures were centrifuged thrice (12,000 rpm) for 15 min. The clear supernatant was used for GC-MS analysis using Agilent 5975C GC-MS system (Agilent Technologies, USA) attached with HP-5 ms Capillary Column (30 m × 0.25 mm i.d. × 0.25 μm film thickness). The machine was equipped with inert MSD triple axis mass detector conditioned at ion trap 200 °C, transfer line 280 °C, electron energy 70 eV (vacuum pressure- 2.21e–0.5 torr) was used for analysis. Helium was used as carrier gas (1 ml/min). Sample volume was 2 μl and injected in a splitless mode. The column temperature was kept at 60 °C for 1 min followed by 5 °C/min up to 250 °C. The major and essential compounds present in samples were identified by their retention times and mass fragmentation patterns using Agilent Chem Station Integrator and the database of National Institute Standard and Technology (NIST) with a MS library version 2010.

### Statistical analysis

All data are reported as mean ± SD of six measurements. Statistical analysis was performed and graphs were prepared using KyPlot Data Analysis and Visualization software version 5.0.2 (32 bit). Comparison between groups were performed using one-way analysis of variance (ANOVA). *p* < 0.05 was considered significant.

## Results

### Acute toxicity study

NOSE and NORE were administered to experimental animals up to 2000 mg/kg dose for evaluation of toxicity and selection of experimental doses. However, up to the highest dose, no signs of mortality or physiological discomfort were observed in the animals. Therefore, 1/40^th^ (50 mg/kg) and 1/10^th^ (200 mg/kg) of the highest dose were selected for the in vivo hepatoprotective experiments.

### Body and liver weight

Significant change (*p* < 0.001) in body weight were observed in CCl_4_ and silymarin groups (Table 1). However, unlike all other groups, final body weight was decreased (17.07 ± 2.56 %) only in case of CCl_4_ group. Similarly, compared to control, significant (*p* < 0.01) difference of liver weight (5.32 ± 0.18 g) in CCl_4_ group was observed, which also resulted in highest relative liver weight (32.53 ± 3.03 g) of the same group. Interestingly, among all groups, both the high dose groups prevented utmost percentage body weight change. However, the relative liver weight of all the groups were comparable, except CCl_4_ group.

**Table 1:** Changes of the body weight (g) and liver weight (g) in different experimental groups. Data represented as mean ± SD of six observations

	Initial body weight	Final body weight	% body weight change	Liver weight	Relative liver weight
Control	22.17 ± 0.70	24.23 ± 0.99*	9.26 ± 1.18 ▲	4.37 ± 0.21	18.04 ± 0.13
CCl_4_	21.10 ± 0.34	17.49 ± 0.27***	17.07 ± 2.56 ▼	5.32 ± 0.18**	32.53 ± 3.03
Silymarin	21.64 ± 0.19	23.43 ± 0.25***	8.29 ± 1.39 ▲	4.30 ± 0.09^NS^	18.37 ± 0.22
NOSE low	21.71 ± 0.49	22.36 ± 0.67^NS^	3.96 ± 3.90 ▲	4.59 ± 0.16^NS^	20.56 ± 1.36
NOSE high	22.25 ± 0.38	22.67 ± 0.18^NS^	1.89 ± 2.38 ▲	4.55 ± 0.29^NS^	20.05 ± 1.18
NORE low	22.14 ± 0.38	22.59 ± 0.21^NS^	2.25 ± 2.02 ▲	4.41 ± 0.16^NS^	19.50 ± 0.58
NORE high	22.20 ± 0.57	22.33 ± 0.46^NS^	1.48 ± 0.26 ▲	4.24 ± 0.10^NS^	19.00 ± 0.52

^NS^ *p* > 0.05, *p < 0.05, **p < 0.01 and ***p < 0.001. Final body weight was compared with initial body weight of corresponding group and liver weight of treated groups were compared with liver weight of control group. ▲ represents increase and ▼ represents decrease

### Liver marker enzymes and biochemical parameters (in vivo)

Table 2 summarized the effect of CCl_4_ and subsequent treatment of silymarin and oleander extracts on the serum enzymatic and biochemical parameters in the experimental animals. The parameters were abnormally altered due to CCl_4_ exposure and subsequently normalized by treatments with silymarin and oleander extracts. Among all the parameters, only the levels of protein and albumin were lowered due to CCl_4_ treatment. The percentage change in the parameters are summarized in Additional file 2.

**Table 2:** Changes in the levels of various enzymatic and biochemical parameters of the serum samples of seven experimental groups. Data represented as mean ± SD of six observations

	Control	CCl_4_	Silymarin	NOSE low	NOSE high	NORE low	NORE high
ACP (K.A.)	3.61 ± 0.31	10.94 ± 1.04***	5.76 ± 0.43** ^b^	10.01 ± 0.61*** ^d^	8.75 ± 0.44*** ^c^	9.64 ± 0.31*** ^d^	8.55 ± 0.32*** ^c^
ALP (K.A.)	10.86 ± 1.29	28.44 ± 1.25***	12.93 ± 0.80^NS a^	24.92 ± 2.27*** ^d^	22.21 ± 2.45** ^a^	24.56 ± 0.95*** ^c^	22.10 ± 1.45*** ^b^
AST (U/ml)	65.88 ± 3.74	137.28 ± 2.95***	74.01 ± 0.16^NS a^	121.27 ± 2.89*** ^a^	99.19 ± 5.48*** ^b^	117.93 ± 6.30*** ^b^	110.25 ± 5.00*** ^b^
ALT (U/ml)	42.63 ± 1.4	126.26 ± 4.88***	53.81 ± 3.15** ^a^	109.27 ± 4.21*** ^c^	81.27 ± 7.66** ^d^	119.23 ± 6.40*** ^d^	95.94 ± 10.39*** ^c^
GGT (U/l)	2.51 ± 0.36	6.06 ± 0.23***	3.35 ± 0.12* ^a^	5.48 ± 0.33*** ^d^	4.9 ± 0.36** ^c^	5.5 ± 0.25*** ^c^	4.48 ± 0.24** ^b^
Glucose (mg/dl)	53.53 ± 1.28	80.38 ± 1.55***	55.23 ± 3.48^NS a^	76.40 ± 4.71** ^d^	63.39 ± 1.42*** ^a^	74.96 ± 2.95*** ^c^	65.86 ± 3.43** ^b^
Protein (g/dl)	4.77 ± 0.29	3.10 ± 0.23**	4.94 ± 0.05^NS a^	3.29 ± 0.21** ^d^	4.19 ± 0.24^NS b^	3.62 ± 0.13** ^c^	3.88 ± 0.07** ^b^
Albumin (g/dl)	3.18 ± 0.07	1.42 ± 0.13***	2.86 ± 0.17* ^a^	1.53 ± 0.27*** ^d^	1.96 ± 0.22*** ^a^	1.55 ± 0.09*** ^NS^	1.71 ± 0.26*** ^NS^
Globulin (g/dl)	1.59 ± 0.30	1.68 ± 0.12^NS^	2.11 ± 0.17^NS c^	1.76 ± 0.28^NS d^	2.23 ± 0.03* ^b^	2.06 ± 0.12^NS c^	2.17 ± 0.20^NS c^
Bilirubin (mg/dl)	0.41 ± 0.03	1.15 ± 0.04***	0.51 ± 0.02* ^a^	1.01 ± 0.03*** ^b^	0.91 ± 0.06*** ^b^	1.11 ± 0.07*** ^d^	1.01 ± 0.02*** ^b^
Urea (mg/dl)	20.58 ± 2.39	111.84 ± 3.54***	35.25 ± 6.78* ^a^	106.83 ± 3.23*** ^d^	75.67 ± 5.59*** ^a^	89.96 ± 3.77*** ^b^	67.71 ± 7.43*** ^a^
LDH (U/l)	219.16 ± 7.19	526.97 ± 7.97***	288.44 ± 10.99*** ^a^	511.31 ± 6.73*** ^d^	433.8 ± 17.47*** ^b^	495.13 ± 12.21*** ^c^	472.24 ± 12.07*** ^b^
Cholesterol (mg/dl)	78.86 ± 3.49	134.52 ± 0.78***	105.62 ± 3.36*** ^a^	122.00 ± 5.39*** ^a^	114.62 ± 5.39*** ^b^	124.96 ± 5.38 *** ^d^	113.48 ± 6.80** ^b^

^NS^p = non-significant (*p* > 0.05), *p < 0.05, **p < 0.01 and ***p < 0.001 vs control

^d^p = non-significant (*p* > 0.05), ^c^ *p* < 0.05, ^b^ *p* < 0.01 and ^a^ *p* < 0.001 vs CCl_4_ group

### Estimation of hepatic anti-oxidative enzymes: catalase and peroxidase

**Fig. 1:**
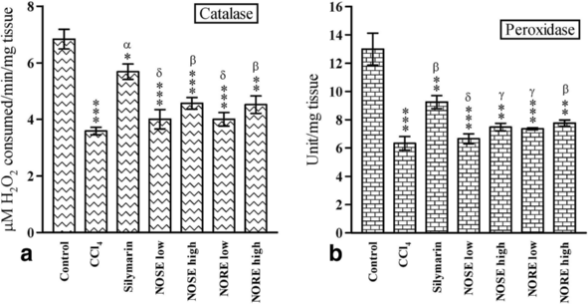
The effect of oleander extracts on the **a** Catalase and **b** Peroxidase activities in CCl_4_ intoxicated liver samples. Data represented as Mean ± SD of six observations. *p < 0.05, **p < 0.01 and ***p < 0.001 Vs control group. δ = *p* > 0.05, γ = *p* < 0.05, β = *p* < 0.01 and α = *p* < 0.001 Vs CCl_4_ group

The activities of catalase and peroxidase enzymes in hepatic tissue were significantly (*p* < 0.001) lowered as a result of CCl_4_ treatment (Fig. 1). The catalase and peroxidase activities in the control group were 6.83 mM H_2_O_2_ consumed/min/mg tissue and 12.99 Unit/mg tissue, which were lowered respectively to 3.58 mM H_2_O_2_ consumed/min/mg tissue and 6.32 Unit/mg tissue due to CCl_4_ administration. This resulted in 47.58 and 51.34 % lowering of antioxidant enzymatic activities, respectively. The percentage change in catalase activities in the silymarin, NOSE high and NORE high groups were 58.56, 27.37 and 25.25, respectively. Similarly, the percentage change in peroxidase levels were 46.20, 18.19 and 22.78, respectively.

### Liver marker enzymes and biochemical parameters (in vitro)

The effects of silymarin and oleander extracts on the in vitro enzymatic and biochemical parameters are summarized in Table 3. The results demonstrated lowering of the parameters as a result of CCl_4_ toxicity. The total protein level was only elevated. However, the elevation was not significant enough (*p* > 0.05). The percentage change of the parameters are enlisted in Additional file 2.

**Table 3:** Changes in the levels of various enzymatic and biochemical parameters of the culture supernatants of the experimental groups. Data represented as mean ± SD of six observations

Parameters (unit)	Control	CCl_4_	Silymarin	NOSE low	NOSE high	NORE low	NORE high
ACP (K.A.)	0.71 ± 0.10	1.74 ± 0.11***	1.17 ± 0.16* ^b^	1.58 ± 0.12*** ^d^	1.48 ± 0.08*** ^c^	1.61 ± 0.15** ^d^	1.40 ± 0.16** ^c^
ALP (K.A.)	3.09 ± 0.11	7.51 ± 0.33***	4.13 ± 0.25** ^a^	6.79 ± 0.24*** ^c^	5.91 ± 0.27*** ^b^	6.99 ± 0.37*** ^d^	6.09 ± 0.50*** ^c^
AST (U/ml)	14.87 ± 0.72	44.49 ± 0.75***	16.39 ± 1.41^NS a^	43.86 ± 2.60*** ^d^	36.32 ± 1.21*** ^a^	38.06 ± 3.76*** ^c^	29.53 ± 3.22** ^b^
ALT (U/ml)	5.81 ± 0.15	27.55 ± 0.52***	10.57 ± 0.29*** ^a^	26.33 ± 1.29*** ^d^	22.17 ± 2.40*** ^c^	24.47 ± 1.82*** ^c^	20.77 ± 1.63*** ^b^
GGT (U/l)	0.59 ± 0.04	0.78 ± 0.02**	0.58 ± 0.01^NS a^	0.77 ± 0.01** ^d^	0.71 ± 0.03* ^d^	0.74 ± 0.00** ^d^	0.72 ± 0.01** ^c^
Bilirubin (mg/dl)	0.13 ± 0.00	0.53 ± 0.05***	0.17 ± 0.00** ^a^	0.56 ± 0.14** ^b^	0.40 ± 0.02*** ^b^	0.465 ± 0.02*** ^d^	0.43 ± 0.02** ^d^
Protein (g/dl)	6.48 ± 0.75	5.3 ± 0.26^NS^	5.7 ± 0.42 ^NS d^	5.61 ± 0.23 ^NS d^	6.05 ± 0.15 ^NS c^	5.77 ± 0.12^NS c^	6.02 ± 0.21^NS c^
LDH (U/l)	35.94 ± 29.66	181.33 ± 11.24**	94.16 ± 7.34* ^a^	138.59 ± 14.05** ^c^	105.00 ± 10.02* ^a^	144.40 ± 11.49** ^c^	114.87 ± 10.53* ^b^

^NS^p = non-significant (*p* > 0.05), *p < 0.05, **p < 0.01 and ***p < 0.001 vs control

^d^p = non-significant (*p* > 0.05), ^c^ *p* < 0.05, ^b^ *p* < 0.01 and ^a^ *p* < 0.001 vs CCl_4_ group

### Lipid peroxidation (MDA content)

**Fig. 2:**
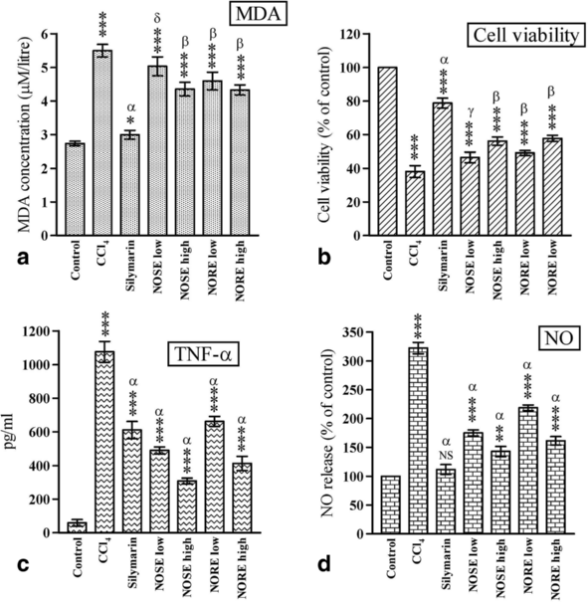
The effects of oleander extract on **a** MDA level, **b** Cell viability, **c** TNF-α level and **d** NO release. Data represented as Mean ± SD of six observations. *p < 0.05, **p < 0.01 and ***p < 0.001 Vs control group. δ = *p* > 0.05, γ = *p* < 0.05, β = *p* < 0.01 and α = *p* < 0.001 Vs CCl_4_ group

The effects of CCl_4_ toxicity and subsequent treatment with plant extracts on the MDA content are represented in Fig. 2a. Initially the MDA content was elevated from 2.73 ± 0.06 μM/l in control to 5.49 ± 0.18 μM/l in CCl_4_ group, contributing to 2.01 fold elevation. However, the subsequent treatments resulted in 45.53, 20.76 and 21.12 % decrease in blood MDA content respectively in silymarin, NOSE high and NORE groups.

### Cell viability

CCl_4_ mediated direct cellular damage (Fig. 2b) was represented by significant (*p* < 0.001) difference (61.92 %) in cell viability between control and CCl_4_ group. However, the cell viability was improved by subsequent treatment with silymarin (*p* < 0.001) and the oleander extracts (*p* < 0.01). The extent of improvement of cell viability were 106.95, 47.32 and 51.60 %, respectively for in silymarin, NOSE high and NORE groups.

### Measurement of TNF-α

The levels of TNF-α in different groups are demonstrated in Fig. 2c. The level of TNF-α in control was 58 ± 20.22 pg/ml, which was 18.54 fold increased to 1075.66 ± 61.53 pg/ml due to CCl_4_ toxicity. However, the TNF-α level was subsequently lowered to 43.19, 71.33 and 61.60 % in silymarin, NOSE high and NORE high groups respectively, compared to CCl_4_ group. All the extract treated groups demonstrated higher TNF-α inhibitory activities than the silymarin group.

### Inhibition of nitric oxide

CCl_4_ toxicity resulted in 3.22 fold increase in NO release compared to control (Fig. 2d). However, significant (*p* < 0.001) lowering of NO level was observed in the treated groups. The NO level in silymarin, NOSE high and NORE high groups were 111.88 ± 8.60, 142.89 ± 8.72 and 161.24 ± 7.39 % respectively, when NO release of control was considered as 100 %. This resulted in 0.34, 0.44 and 0.50 fold lowering of NO level.

### Histopathological studies

**Fig. 3:**
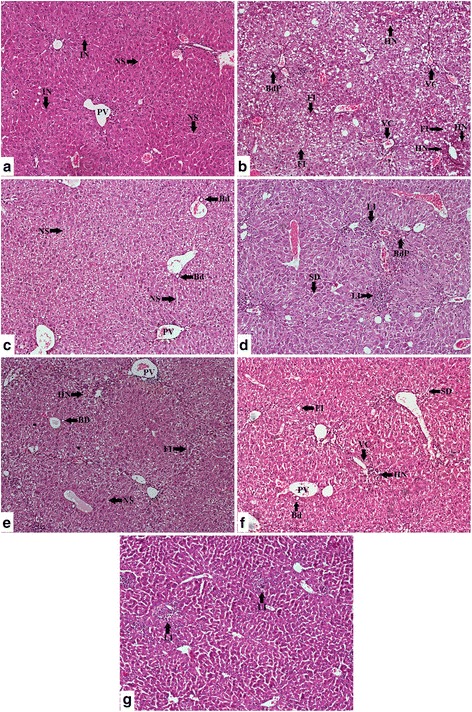
Photomicrographs (100×) of the histopathological examinations of the liver samples of different groups. Even though the extract treated groups possessed injury marks however, the extent of signs of injury were much lower in the extract treated groups compared to CCl_4_ group. **a** Control group liver demonstrated normal liver architecture with normal sinusoids (NS), hepatocytes with intact nucleus (IN), un-inflamed portal vein (PV). **b** CCl_4_ group liver demonstrated significant loss of hepatocellular architecture with extensive fatty infiltration (FI) leading to steatosis, bile duct proliferation (BdP), vascular congestion (VC) and haemorrhagic necrosis (HN) around portal vein. Loss of hepatic nodular structure and disorganized hepatocytes marked the CCl_4_ induced liver damage. **c** Silymarin group demonstrated hepatoprotective activity by substantial amendment of proliferated bile duct (Bd) with normal sinusoids (NS) and intact portal veins (PV). **d** NOSE low group was marked by less leukocyte infiltrations (LI), sinusoidal dilations (SD) and bile duct proliferation (BdP). **e** NOSE high group reflected comparatively less haemorrhagic necrosis (HN) and fatty infiltrations (FI). **f** NORE low group also showed traces of sinusoidal dilations (SD), haemorrhagic necrosis (HN) and fatty infiltration (FI). **g** NORE high group demonstrated lowering of most of the injury signs however, leukocyte infiltrations (LI) could be identified in the liver samples

**Fig. 4:**
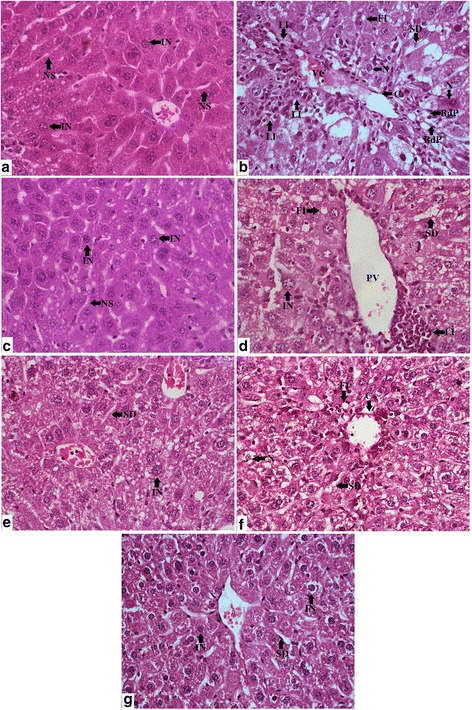
Photomicrographs (400×) of the histopathological examinations of the liver samples of different groups. **a** Control group liver sampled possessed well packed hepatocytes with intact nucleus (IN) and normal sinusoids (NS). **b** CCl_4_ group liver possessed extensive fatty infiltrations (FI), Necrotic hepatocytes (N), prominent signs of inflammation with leukocyte infiltrations (LI), prominent calcification (C) around the congested vesicles (VC) with bile duct proliferations (BdP). **c** Silymarin group liver samples were characterized with normal sinusoids (NS) and intact nucleus (IN) containing healthy hepatocytes. **d** NOSE low group demonstrated lower fatty infiltrations (FI), sinusoidal dilations (SD) and leukocyte infiltrations (LI). **e** NOSE high group resulted in renewal of normal hepatic architecture with several hepatocytes with intact nucleus (IN) and lowered sinusoidal dilations (SD). **f** NORE low group livers possessed few necrotic (N) cells, fatty infiltrations (FI) and zonal calcification (C) around the portal veins (PV). **g** NOSE high group showed near to normal hepatic architecture with predominantly intact nucleus (IN) containing normal hepatocytes and undiluted normal sinusoids (NS)

The extent of liver damage caused by haloalkane toxicity was clearly evident through the haematoxylin-eosin staining in the histopathological studies (Figs. 3 and 4). The normal hepatic architecture in the control could be described by presence of intact spherical nucleus, healthy cellular architecture, well preserved hepatocellular symmetry, marked cellular boundaries, and clear cytoplasm. Extensive hepatocellular damage in the CCl_4_ group could be described through the presence of dilated sinusoids, fatty infiltration and deformed cellular margins. Extensive sings of inflammation was visible in terms of infiltration of leukocytes in the CCl_4_ group. Moreover, vascular congestions and scattered regions of hepatic fibrosis marked the intensive CCl_4_ mediated toxicity. The signs of hepatic damage were also visible in the silymarin and extract treated groups, however the extent of damage was much lower than the CCl_4_ group. Liver samples of NOSE and NORE high groups possessed much lover frequency of necrotic nucleus, dilated sinusoids, infiltrated leukocytes, deformed cellular architecture and vascular congestions. This demonstrated amelioration of haloalkane induced liver damage by the oleander extracts.

### FTIR and GC-MS analysis

**Fig. 5:**
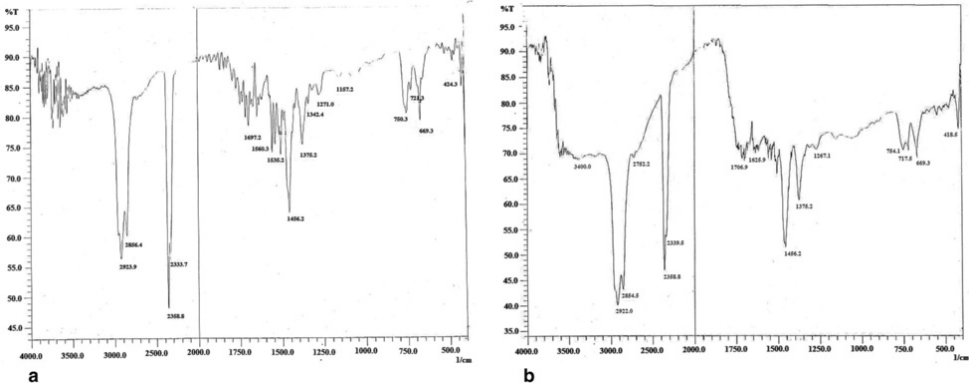
Fourier transform infrared spectra of **a** NOSE and **b** NORE

FTIR analysis of NOSE and NORE (Fig. 5) were performed to identify the predominant chemical groups present in the extracts. The IR spectrums displayed different peaks (Additional file 3) at corresponding to different functional groups [24].

**Fig. 6:**
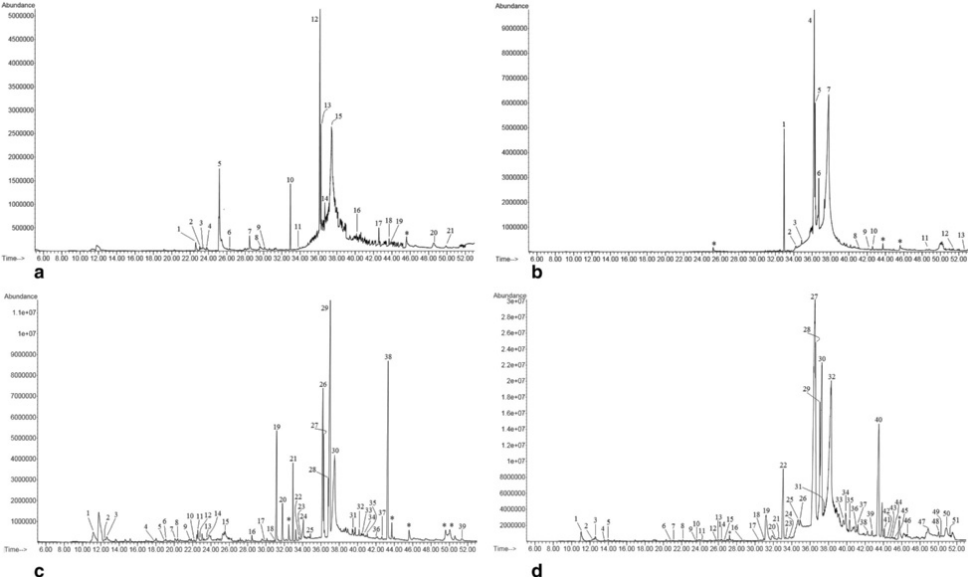
Gas chromatographic (GC) spectra of NOSE and NORE. **a** Dichloromethane fraction of NOSE, **b** N-hexane fraction of NOSE, **c** Dichloromethane fraction of NORE and **d** N-hexane fraction of NORE. * represents repeat compound/unidentified compound/column component

NOSE and NORE were bi-fractionated and subjected to GC-MS analysis. The analysis identified (Fig. 6 and Additional file 4) several bioactive constituents in the oleander extracts such as vanillin, trans- isoeugenol, murrayafoline A, apocynin, squalene, β-amyrin, lupeol, tocopherol etc.

## Discussion

The toxicity profile of CCl_4_ has been thoroughly studied and is well established [25–27]. CCl_4_ has a rich history of environmental toxicity and occupational hazard due to its extensive use in industrial sectors. This had led to an awareness in the industrial and domestic use of CCl_4_ from the early 70’s, leading to a ban on production and import of CCl_4_ in 1996 [28]. However, CCl_4_ continues to play an essential role as a model haloalkane xenobiotic in the pharmaco-toxicological studies. CCl_4_ induced liver damage is primarily initiated by the formation of trichloromethyle radical (CCl_3_^●^) due to cytochrome P450 mediated reductive dehalogenation of CCl_4_ [8, 29]. Persistent formation of the highly reactive CCl_3_^●^ in the liver cause steatosis, lipid peroxidation leading to hepatocarcinoma [8]. Additionally, fusion of CCl_3_^●^ with diatomic oxygen generates highly reactive trichloromethylperoxy radical (CCl_3_OO^●^) which further causes damage to mitochondria, endoplasmic reticulum and plasma membrane permeability, resulting in loss of cytosolic Ca^2+^ sequestration and homeostasis [8]. The present study demonstrated that, antioxidant rich oleander extracts hold the potentiality to ameliorate CCl_4_ induced hepatotoxicity in murine model. The data were further supported by the identification of several phytochemical constituents with well-established hepatoprotective potentialities.

CCl_4_ toxicity caused significant (*p* < 0.001) loss of body weight as well as highest relative liver weight in the animals. Compared to control and silymarin group, the final body weight change in the extract treated groups were much less. This may presumably indicate the potentiality of oleander extracts to restrict body weight increase through anti-hyperlipidemic activity as demonstrated previously by body weight lowering capability of oleander [30]. Compared to control, 1.80 fold higher relative liver weight in the CCl_4_ group reflected formation of fatty liver which was further supported by increased blood cholesterol level and extensive steatosis throughout the CCl_4_ intoxicated liver samples. However, comparatively lower relative liver weight associated with insignificant total body weight change demonstrated the potentiality of oleander extracts to prevent severe physiological consequences due to CCl_4_ poisoning.

The altered levels of hepatobillary enzymes transaminase and phosphatase along with other enzymatic and biochemical parameters serve as biomarkers of hepatotoxicity whereas, normalization of the parameters represents improvement of normal liver functions [31–33]. *N. indicum* leaf extract has previously been shown to normalize similar liver biomarkers under CCl_4_ toxicity [14]. Moreover, oleander floral extracts normalized the elevated AST, GGT, ALT, LDH, ALP and creatinine levels in blood under isoproterenol-induced oxidative stress in rats [34]. Furthermore, the anti-hyperlipidemic activities were evident through lowering of cholesterol levels and associated lowering of fatty infiltrations and steatosis under the histopathological studies, which were further supported by the previous studied of Gayathri et al. [30]. The blood glucose level lowering capacity of the oleander extracts is also supported by its ethnopharmacological claim of hypoglycaemic activities as well as a recent study that demonstrated anti-diabetic activities of oleander leaf under alloxan induced diabetes in mouse [35]. CCl_4_ toxicity in the present study, resulted in significant (*p* < 0.001) elevation of ACP, ALP, AST, ALT, GGT, LDH, glucose, urea, globulin, bilirubin and cholesterol levels, signifying liver injury. Only the protein and albumin levels were decreased compared to control. The parameters were subsequently normalized up to certain extent due to silymarin and oleander extract treatments.

Oleander extracts were further evaluated through primary culture to ameliorate CCl_4_ induced toxicity in mouse hepatocytes in vitro. Cultured liver cells are biochemically prototypical to intact hepatic system and therefore, serves as a model for evaluation of in vitro hepatotoxicity [8]. The results of in vitro studies demonstrated hepatoprotective potentialities of the extracts by normalization of the liver enzymatic and biochemical parameters as well. Liver, in spite of having the highest regenerative capacity, persistent exposure to cytochrome P450 activated organic xenobiotics and their reactive metabolic intermediates [36] cause tremendous hepatocellular necrosis. Necrotic hepatocytes with fragmented nucleus and distorted cellular margins were clearly evident through histopathological observations. Zonal haemorrhagic necrosis around portal veins in the CCl_4_ group signified that the hepatocellular injury had exceeded the regenerative power of the liver. This was further confirmed through MTT cell viability assay which showed 61.92 % loss of cell viability due to direct CCl_4_ exposure in the culture medium. However, subsequent treatment with silymarin and oleander extracts prevented further hepatocellular damage resulting in significantly (*p* < 0.001) increase in viable cells compared to CCl_4_ group.

Xenobiotic intoxicated liver encounters a plethora of free radicals and reactive metabolic intermediates which results in great damage to cellular components and the extent of free radical medicated damage is proportional to the extent of lipid peroxidation [37–39]. The resultant oxidative stress further deactivates the cellular antioxidative enzymes [40]. Peroxidase and catalase are two major antioxidative enzymes responsible for the neutralization of free radicals. Peroxidase reduces and converts hydroperoxides and lipid peroxides into non-reactive species, whereas catalase prevents the formation of highly reactive OH^●^ by scavenging H_2_O_2_ which participates in the Fenton reaction. Inhibition of both enzymatic activities accumulates O_2_^●−^ and H_2_O_2_ which accentuate a cascade of free radical formation. CCl_4_ derived trichloromethylperoxy radical (CCl_3_OO^●^) abstracts proton from polyunsaturated fatty acids in the biological membranes and cause lipid peroxidation [8]. The reactive carbonyl compound MDA is a biomarker for oxidative damage and subsequent lipid peroxidation. The 70 % hydro-methanolic extracts of oleander previously demonstrated potent free radical scavenging activities by inhibiting some of the most harmful free radicals such as OH^●^, O_2_^●−^, NO, ONOO^−^, H_2_O_2_ and ^1^O_2_ [11]. The study further showed that oleander extracts hold the ability to prevent Fenton reaction by means of neutralization of hydroperoxides and also inhibit lipid peroxidation by scavenging OH^●^ directly. Lipid membranes are vulnerable to oxidative and nitrosative stress due to high amount of polyunsaturated fatty acids and transition metals. Transition metals such as iron are capable of damaging nuclear proteins, DNA, inhibit enzymes and degrade lipid membrane through oxidative Haber-Weiss reaction [41–43]. Our previous study showed potent iron chelation ability by the oleander extracts [11]. Recent studies also showed that 70 % hydro-methanolic extract of *N. indicum* leaf could ameliorate hemochromatosis and fibrosis in liver due to its potent antioxidant and iron chelation activities [13, 14]. Thus, the present study corroborates the previous findings of the antioxidative protection by oleander extracts. Lowering of liver antioxidative enzymes due to CCl_4_ toxicity marked increased oxidative stress, resulting in the elevation of lipid peroxidation. However, the diminished catalase and peroxidase levels and elevated MDA level were subsequently normalized by NOSE and NORE administration. The present observations thus, remains in accordance with the previous reports showing improvement of in-house antioxidative enzymes by oleander leaf and flower extracts under oxidative stress [34, 44].

Liver diseases are accompanied by inflammatory conditions which accentuate the progression of liver failure. TNF-α and NO play a major role as pro-inflammatory mediators during CCl_4_ mediated liver injury which leads towards apoptotic cell death and fibrosis [8, 45]. Under such xenobiotic induced hepatotoxic conditions, activated Kupffer cells secret a vast array of cytokines (TNF-α, IL-1, IL-8, IL-6), chemokines (KC/GRO, IP-109, MIP-2, MCP-1) and pro-inflammatory mediators such as NO, which initiate hepatic inflammation and induce toxicity either by direct cellular damage or by chemoacttracting neutrophils and lymphocytes [46]. TNF-α initiates hepatotoxicity and fibrogenesis and overproduction of NO results in endotoxin shock and inflammatory hepatic injury [45, 47, 48]. Inducible nitric oxide synthase (iNOS) in liver parenchymal and non-parenchymal cells are induced by TNF-α resulting in nitrosative stress. In liver mitochondria, O_2_^●−^ couples with an excess of NO to generate the highly reactive ONOO^−^. Interestingly, the anti-inflammatory activity of oleander extract is well known in traditional medicine and the extracts are also used in the treatment of diverse inflammatory disorders [17, 49]. In a preliminary study, Erdemoglu et al. [50] demonstrated anti-inflammatory activities of ethanolic and aqueous extracts of oleander leaves. Moreover, the 70 % hydro-methanolic extract of oleander leaves were demonstrated to possess anti-inflammatory activities by modulation of Th1/Th2 cytokine levels accompanied by inhibition of NO, COX and PGE_2_ levels in concanavalin A activated murine splenic lymphocytes [15, 16]. In the present study, CCl_4_ toxicity resulted significant (*p* < 0.001) increase in TNF-α and NO levels, which were subsequently lowered by the oleander extracts. This exhibited potent anti-inflammatory activities of oleander extracts through suppression of pro-inflammatory mediators of chronic hepatotoxicity.

The hepatoprotective potentiality of oleander extracts were further established through detailed histopathological studies. The results clearly demonstrated that deformed hepatic architecture due to CCL_4_ toxicity was subsequently attenuated by oleander extracts. CCl_4_ toxicity initiated tremendous hepatocellular degeneration as evident by scattered hepatocellular necrosis and in some cases with central haemorrhagic necrosis. Deformed nucleus containing overlapping hepatocytes with irregular cellular margins were characteristics of CCl_4_ group which highly contrasted the prominent nucleus containing healthy hepatocytes with conspicuous cellular boundaries in the control. Loss of cellular integrity in the CCl_4_ group were visible in terms of disorganized arrangement of the hepatocytes. Interestingly, liver samples of the CCl_4_ group exhibited an extensive, yet typical signs of inflammation by means of infiltration of leukocytes from the portal regions extended towards the hepatocyte rich central zones, which were comparatively less in the silymarin and extract treated groups. Extensive fatty infiltration throughout the CCl_4_ group liver samples signified initiation of macrovascular steatosis and adipose degeneration that were less in the NOSE and NORE groups. Moreover, the deformity of the hepatic nodules associated vascular congestions with occasional bile duct proliferations were also lowered in the silymarin and extract treated groups. Overall, the deformed hepatocellular architecture of the CCl_4_ group were significantly normalized by oleander extracts.

NOSE and NORE were further analysed using FTIR and GC-MS method to reveal the phytochemical constituents responsible for the potent hepatoprotective potentiality. Different functional groups were identified through FTIR analysis, which correlating with the compounds identified in the GC-MS study. Alkanes like tetradecane and hexadecane resulted in = C-H bending. The C-O stretching of carboxylic moiety resulted due to the presence of linoleic acid, n-hexadecanoic acid, benzoic acid, eicosenoic acid, oleic acid etc. The O-H stretching of the alcohol/phenolic species were supported by the presence of lupeol, amyrin, isoeugenol, vanillin and other phenolic phytochemicals. In GC-MS analysis, both the fractions of the extracts revealed the presence of several bioactive constituents which corroborates a previous report of a preliminary phytochemical analysis of oleander [51]. The 70 % hydro-methanolic extracts of oleander leaf, steam and root possessed high amount of phenolic and flavonoid contents, contributing to its antioxidant and free radical scavenging activities [11]. A detailed account of related bioactivities of individual phytocompounds are enlisted in Additional file 5, which supports the view that the hepatoprotective activity of oleander extract resulted due to additive and synergistic activities of the component phytochemicals.

## Conclusion

**Fig. 7:**
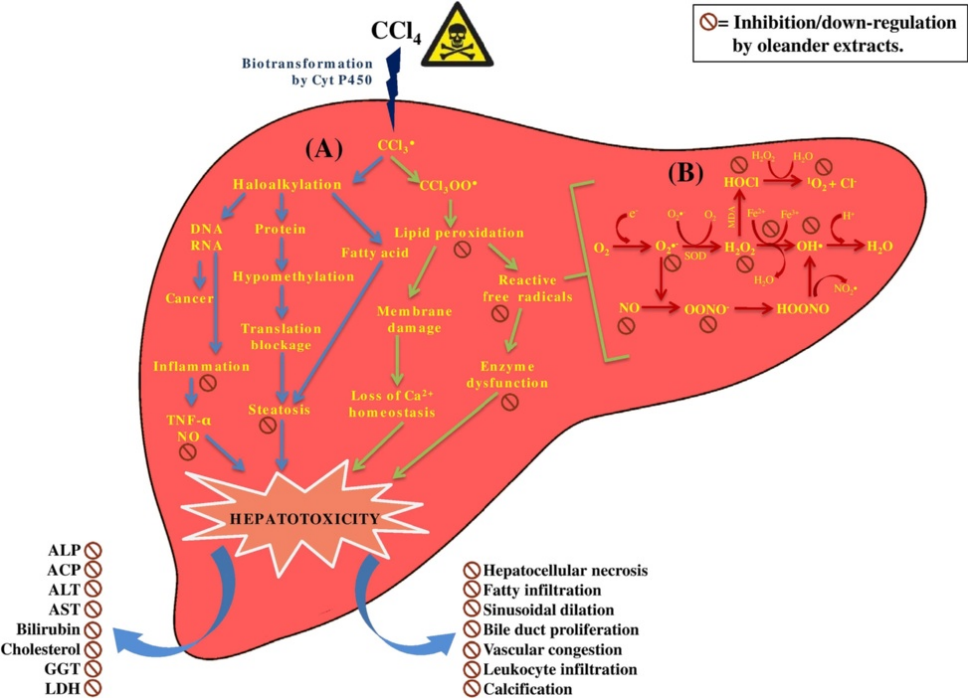
Schematic representation of the mode of action of oleander extracts to ameliorate CCl_4_ induced hepatotoxicity. Section (A) demonstrates the mechanism of CCl_4_ induced hepatotoxicity which is predominantly mediated by oxidative stress and inflammatory injury due to the formation of reactive metabolic intermediates. Cyt P450 = cytochrome P450, CCl_3_^●^ = trichloromethyle radical, CCl_3_OO^●^ = trichloromethylperoxy radical, TNF-α = tumor necrosis factor-α, NO = nitric oxide. Section (B) represents the free radical formation cascade during xenobiotic induced hepatotoxicity causing oxidative and nitrosative stress. CCl_4_ induced hepatic damage distorts normal liver architecture and leads to leakage of liver marked enzymes into the blood stream. OH^●^ = hydroxyl radical, O_2_^●−^ = superoxide radical, MPO = myeloperoxidase, ONOO^−^ = peroxynitrite, HOONO = peroxynitrous acid, NO_2_^●^ = nitrogen dioxide radical, ^1^O_2_ = singlet oxygen, H_2_O_2_ = hydrogen peroxide, SOD = Superoxide dismutase, HOCl = hypochlorous acid

The present study demonstrated that oleander stem and root extracts attenuated reactive species mediated liver damage which was evident through significant lowering of lipid peroxidation and elevation of antioxidant enzymatic activities. Normalization of the liver enzymatic and biochemical markers under both in vivo and in vitro liver function tests provided convincing evidence of the hepatoprotective potentialities of the oleander extracts. Histopathological studies provided confirmative visual support towards the improvement of hepatic architecture as well. Therefore, it may be concluded that oleander extracts ameliorated CCl_4_ induced liver injury primarily through normalization of liver antioxidant enzymes and inhibition of pro-inflammatory signals. The hepatoprotective activities may be attributed to the presence of bioactive constituents in the extracts. The mode of hepatoprotective activity of oleander may thus, be represented as Fig. 7. Our future endeavour will be isolation and characterization of major bioactive species and detailed study of their physiological implications.

## Abbreviations

ACP, acid phosphatase; ALP, alkaline phosphatase; ALT, alanine transaminase; AST, aspartate transaminase; CCl_4_, carbon tetrachloride; DW, dry weight; FTIR, Fourier Transform Infrared; GC-MS, Gas chromatography-mass spectrometry; GGT, gamma-glutamyl transferase; LDH, lactate dehydrogenase; MDA, malondialdehyde; MTT, 3-(4,5-dimethylthiazol-2-yl)-2,5-diphenyltetrazolium bromide; NO, nitric oxide; NORE, *Nerium oleander* root extract; NOSE, *Nerium oleander* stem extract; SDS, sodium dodecyl sulphate; TBARS, thiobarbituric acid reactive substances; TNF-α, tumor necrosis factor-α.

## Additional files

Additional file 1: Preparation of 70 % hydro-methanolic extract of oleander stem and root. (DOCX 153 kb) Additional file 2: Summarizes the percentage change of different enzymatic and biochemical parameters in the serum samples and culture medium. (DOCX 16 kb) Additional file 3: Fourier transform infrared spectroscopy peak values of NOSE and NORE corresponding to Fig. 5. (DOCX 17 kb) Additional file 4: Chemical fingerprint of dichloromethane fraction of NOSE corresponding to Fig. 6a. Chemical fingerprint of n-hexane fraction of NOSE corresponding to Fig. 6b. Chemical fingerprint of dichloromethane fraction of NORE corresponding to Fig. 6c. Chemical fingerprint of n-hexane fraction of NORE corresponding to Fig. 6d. (DOCX 29 kb) Additional file 5: Bioactivities of identified compounds. ▲ means increase; ▼ means decrease; ♦ means no change. (DOCX 33 kb)
