# Supplementary figures and images for: Haloalkane induced hepatic insult in murine model: amelioration by Oleander through antioxidant and anti-inflammatory activities, an in vitro and in vivo study
Source: BMC Complement Altern Med. 2016 Aug 11;16:280. doi: 10.1186/s12906-016-1260-4 (PMC4982413; doi:10.1186/s12906-016-1260-4)

**Additional file 1**

Preparation of 70% hydro-methanolic extract of oleander stem and root.


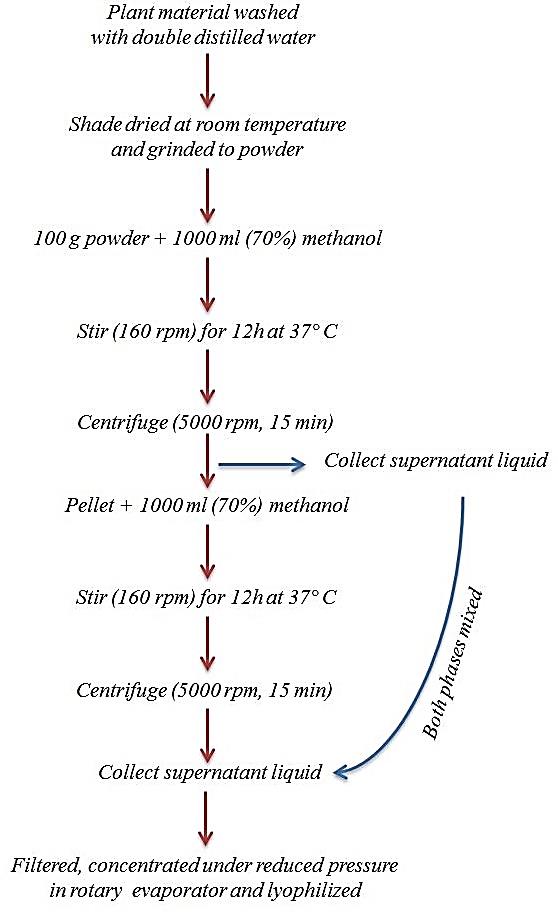

Supplement: Additional file 1: — Preparation of 70 % hydro-methanolic extract of oleander stem and root. (DOCX 153 kb) [file 12906_2016_1260_MOESM1_ESM.docx]
