# Supplementary material for: Haloalkane induced hepatic insult in murine model: amelioration by Oleander through antioxidant and anti-inflammatory activities, an in vitro and in vivo study
Source: BMC Complement Altern Med. 2016 Aug 11;16:280. doi: 10.1186/s12906-016-1260-4 (PMC4982413; doi:10.1186/s12906-016-1260-4)
Supplement: Additional file 2: — Summarizes the percentage change of different enzymatic and biochemical parameters in the serum samples and culture medium. (DOCX 16 kb) [file 12906_2016_1260_MOESM2_ESM.docx]

**Additional file 2**

Summarizes the percentage change of different enzymatic and biochemical parameters in the serum samples and culture medium.

|  | | **Silymarin** | **NOSE low** | **NOSE high** | **NORE low** | **NORE high** |
| --- | --- | --- | --- | --- | --- | --- |
|  | ***In vivo* experiments** | | | | | |
| **ACP** | | 47.34▲ | 8.50▲ | 20.01▲ | 11.88▲ | 21.84▲ |
| **ALP** | | 54.53▲ | 12.37▲ | 21.90▲ | 13.64▲ | 22.29▲ |
| **AST** | | 46.08▲ | 11.66▲ | 27.74▲ | 14.09▲ | 19.68▲ |
| **ALT** | | 57.38▲ | 13.45▲ | 35.63▲ | 5.56▲ | 24.01▲ |
| **GGT** | | 44.71▲ | 9.57▲ | 19.14▲ | 9.24▲ | 26.07▲ |
| **Glucose** | | 31.28▲ | 4.95▲ | 21.13▲ | 6.74▲ | 18.06▲ |
| **Protein** | | 59.35▼ | 6.12▼ | 35.16▼ | 16.77▼ | 25.16▼ |
| **Albumin** | | 101.40▼ | 7.74▼ | 38.02▼ | 9.15▼ | 20.42▼ |
| **Globulin** | | 25.59▼ | 4.76▼ | 32.73▼ | 22.61▼ | 29.16▼ |
| **Bilirubin** | | 55.65▲ | 12.17▲ | 20.86▲ | 3.47▲ | 12.17▲ |
| **Urea** | | 68.48▲ | 4.47▲ | 32.34▲ | 19.56▲ | 39.45▲ |
| **LDH** | | 45.26▲ | 2.97▲ | 17.68▲ | 6.04▲ | 10.38▲ |
| **Cholesterol** | | 21.48▲ | 9.30▲ | 14.79▲ | 7.10▲ | 15.64▲ |
| ***In vitro* experiments** | | | | | | |
| **ACP** | | 32.75▲ | 9.19▲ | 14.94▲ | 4.59▲ | 19.54▲ |
| **ALP** | | 45.00▲ | 9.58▲ | 21.30▲ | 6.92▲ | 18.90▲ |
| **AST** | | 63.16▲ | 1.41▲ | 18.36▲ | 14.45▲ | 33.62▲ |
| **ALT** | | 61.63▲ | 4.42▲ | 19.52▲ | 11.17▲ | 24.60▲ |
| **GGT** | | 25.64▲ | 1.28▲ | 8.97▲ | 5.12▲ | 7.69▲ |
| **Bilirubin** | | 67.92▲ | 5.66▼ | 24.52▲ | 13.20▲ | 18.86▲ |
| **Protein** | | 7.54▼ | 5.84▼ | 14.15▼ | 8.86▼ | 13.58▼ |
| **LDH** | | 48.07▲ | 23.57▲ | 42.09▲ | 20.36▲ | 36.65▲ |

▲ represents increase and ▼ represents decrease.
