# Supplementary material for: Haloalkane induced hepatic insult in murine model: amelioration by Oleander through antioxidant and anti-inflammatory activities, an in vitro and in vivo study
Source: BMC Complement Altern Med. 2016 Aug 11;16:280. doi: 10.1186/s12906-016-1260-4 (PMC4982413; doi:10.1186/s12906-016-1260-4)
Supplement: Additional file 3: — Fourier transform infrared spectroscopy peak values of NOSE and NORE corresponding to Fig. 5. (DOCX 17 kb) [file 12906_2016_1260_MOESM3_ESM.docx]

**Additional file 3**

**Fourier transform infrared spectroscopy peak values of NOSE and NORE corresponding to Fig. 5.**

| **Wave number (cm^-1^)** | **Type of Bonds** | **Functional groups** | **Type of**  **Vibration** |
| --- | --- | --- | --- |
| 3400.0 | O-H | Alcohol/Phenol | Stretching |
| 2923.9 | CH_2_-O | Alkane (C-H) Aliphatic | Stretching |
| 2922.0 |  |  |  |
| 2856.4 | CH-pyrrole | Alkane (C-H) | Stretching |
| 2854.5 |  |  |  |
| 2752.2 | =C-H | Acid | Stretching |
| 2358.8 | C=C | ---- | Stretching |
| 2333.7 | C=O | Aliphatic ketone | ---- |
| 1706.9 | C=O | Carbonyl/ Acid | Stretching |
| 1697.2 |  |  |  |
| 1625.9 | C-C | Alkene | Stretching |
|  | N-H | Amide | Bending |
| 1560.3 | N-O | Nitro | Stretch |
|  | N-H | Amide | Bending |
| 1535.2 | N-H | Amide | Bending |
| 1456.2 | C=C | Aromatic | Stretching |
|  | CH_3_ | Alkane (C-H) | Bending |
| 1375.2 | -C-H | Alkane | Bending |
|  | N-O | Nitro | Stretching |
| 1342.4 | C-N | Amine (aryl) | Stretching |
| 1271.0 | C-O | Carboxylic acid | Stretching |
| 1267.1 |  |  |  |
| 1157.2 | C-O | Ester | Stretching |
| 754.1 | =C-H | Alkene | Bending |
| 750.3 |  |  |  |
| 721.3 |  |  |  |
| 717.5 |  |  |  |
| 669.3 |  |  |  |
| 424.3 | N-H | Amide | Bending |
| 418.5 |  |  |  |
