# Supplementary material for: Haloalkane induced hepatic insult in murine model: amelioration by Oleander through antioxidant and anti-inflammatory activities, an in vitro and in vivo study
Source: BMC Complement Altern Med. 2016 Aug 11;16:280. doi: 10.1186/s12906-016-1260-4 (PMC4982413; doi:10.1186/s12906-016-1260-4)
Supplement: Additional file 4: — Chemical fingerprint of dichloromethane fraction of NOSE corresponding to Fig. 6a. Chemical fingerprint of n-hexane fraction of NOSE corresponding to Fig. 6b. Chemical fingerprint of dichloromethane fraction of NORE corresponding to Fig. 6c. Chemical fingerprint of n-hexane fraction of NORE corresponding to Fig. 6d. (DOCX 29 kb) [file 12906_2016_1260_MOESM4_ESM.docx]

**Additional file 4**

**Chemical fingerprint of dichloromethane fraction of NOSE corresponding to Figure 6A.**

| **Sl No.** | **Name of compounds** | **Retention time (min)** | **Relative abundance (%)** |
| --- | --- | --- | --- |
|  | trans-Isoeugenol | 22.602 | 0.4178 |
|  | Ethanone, 1-(2-hydroxyphenyl)- | 23.152 | 0.2176 |
|  | Methylparaben | 23.352 | 0.2916 |
|  | Apocynin | 23.796 | 0.2499 |
|  | Ethanone, 1-(2,4-dihydroxyphenyl)- | 25.191 | 3.9336 |
|  | 3,4-Dihydroxyacetophenone | 26.061 | 0.1377 |
|  | Phenol, 2,6-dimethoxy-4-(2-propenyl)- | 28.482 | 0.6191 |
|  | 4-((1E)-3-Hydroxy-1-propenyl)-2-methoxyphenol | 29.601 | 0.4895 |
|  | 2-Pentanone, 1-(2,4,6-trihydroxyphenyl) | 30.108 | 0.2385 |
|  | Hexadecanoic acid, methyl ester | 32.929 | 1.205 |
|  | Pentadecanoic acid | 33.980 | 0.1802 |
|  | Methyl 9-cis,11-trans-octadecadienoate | 36.163 | 0.1802 |
|  | trans-13-Octadecenoic acid, methyl ester | 36.257 | 3.0331 |
|  | Methyl stearate | 36.689 | 2.2393 |
|  | 9,12-Octadecadienoic acid (Z,Z)- | 37.452 | 21.6783 |
|  | Linoleic acid ethyl ester | 41.042 | 0.4069 |
|  | Tryptamine | 42.575 | 1.2043 |
|  | 5-Dodecyne | 43.926 | 0.4615 |
|  | 13-Tetradece-11-yn-1-ol | 44.039 | 0.3528 |
|  | (+)-.gamma.-Tocopherol, O-methyl- | 48.555 | 0.7831 |
|  | Dodeca-1,6-dien-12-ol, 6,10-dimethyl | 49.900 | 0.2021 |

**Chemical fingerprint of n-hexane fraction of NOSE corresponding to Figure 6B.**

| **Sl No.** | **Name of compound** | **Retention time (min)** | **Relative abundance (%)** |
| --- | --- | --- | --- |
|  | Hexadecanoic acid, methyl ester | 32.960 | 3.4617 |
|  | n-Hexadecanoic acid | 34.224 | 0.5718 |
|  | Heptadecanoic acid, methyl este | 34.856 | 1.3692 |
|  | 9,12-Octadecadienoic acid (Z,Z)-, methyl ester | 36.232 | 21.2314 |
|  | 9,12,15-Octadecatrienoic acid, methyl ester, (Z,Z,Z)- | 36.326 | 6.7936 |
|  | Methyl stearate | 36.720 | 6.6128 |
|  | 9,12-Octadecadienoic acid (Z,Z)- | 37.802 | 35.7523 |
|  | E,E-10,12-Hexadecadien-1-ol | 41.080 | 0.4201 |
|  | 9-Eicosyne | 42.125 | 0.3261 |
|  | 1H-Indole, 4-methyl- | 42.594 | 0.2528 |
|  | Vitamin E | 48.480 | 0.1049 |
|  | 5-Dodecyne | 51.395 | 0.1613 |
|  | Supraene | 52.633 | 0.0156 |

**Chemical fingerprint of dichloromethane fraction of NORE corresponding to Figure 6C.**

| **Sl. No.** | **Name of compounds** | **Retention time (min)** | **Relative abundance (%)** |
| --- | --- | --- | --- |
|  | 1,3-Dioxane, 2-pentadecyl- | 11.761 | 5.2667 |
|  | 2-Propenoic acid, octyl ester | 12.437 | 0.1816 |
|  | 3-Hexene, 3-ethyl-2,5-dimethyl- | 12.643 | 0.3811 |
|  | 2-Nonanone | 17.891 | 0.1263 |
|  | Propanamide, N-methyl- | 18.955 | 0.1545 |
|  | 4-Hydroxy-3-methylacetophenone | 19.086 | 0.1436 |
|  | Phenol, 2,6-dimethoxy- | 20.074 | 0.1332 |
|  | 2-Butanone, 4,4-dimethoxy- | 20.394 | 0.1367 |
|  | Vanillin | 21.763 | 0.036 |
|  | Nonanoic acid, 9-oxo-, methyl este | 22.139 | 0.0842 |
|  | Trans-Isoeugenol | 22.564 | 0.6153 |
|  | Phenol, 2-methoxy-4-(1-propenyl)- | 22.708 | 0.0641 |
|  | Acetophenone, 4'-hydroxy- | 23.258 | 0.0829 |
|  | 9-Oxononanoic acid | 23.972 | 0.2967 |
|  | Ethanone, 1-(2,4-dihydroxyphenyl)- | 25.542 | 0.9568 |
|  | Phenol, 2,6-dimethoxy-4-(2-propenyl)- | 28.463 | 0.2397 |
|  | 4-((1E)-3-Hydroxy-1-propenyl)-2-methoxyphenol | 29.583 | 0.2492 |
|  | Pentadecanoic acid, methyl ester | 30.927 | 0.048 |
|  | 7-Octen-2-ol, 2,6-dimethyl- | 31.178 | 5.6341 |
|  | Benzene, 1-methoxy-3-(3-methyl-3-butenyl)- | 31.803 | 1.5863 |
|  | Hexadecanoic acid, methyl ester | 32.948 | 2.3541 |
|  | 4-Methylcarbazole | 33.598 | 0.2204 |
|  | 1-Methylcarbazole | 33.711 | 0.0971 |
|  | n-Hexadecanoic acid | 34.086 | 1.7128 |
|  | Heptadecanoic acid, methyl ester | 34.843 | 0.2046 |
|  | 9,12-Octadecadienoic acid (Z,Z)-,methyl ester | 36.219 | 9.0872 |
|  | 9-Octadecenoic acid, methyl ester,(E)- | 36.301 | 3.4019 |
|  | Methyl stearate | 36.807 | 1.6631 |
|  | Murrayafoline A | 37.026 | 18.4853 |
|  | 9,12-Octadecadienoic acid (Z,Z)- | 37.496 | 14.9234 |
|  | Methyl 9-eicosenoate | 39.735 | 0.2174 |
|  | Methyl 18-methylnonadecanoate | 40.160 | 0.2174 |
|  | Bicyclo[10.1.0]tridec-1-ene | 40.398 | 0.114 |
|  | 2-Methyl-Z,Z-3,13-octadecadienol | 40.504 | 0.1163 |
|  | 1-Pentadecyne | 41.055 | 0.1188 |
|  | 13-Tetradece-11-yn-1-ol | 42.143 | 0.1317 |
|  | 1H-Indole, 5-methyl- | 42.644 | 0.1365 |
|  | Acetophenone oxime, 3,5-di(tert-butyl)-4-hydroxy- | 43.332 | 11.028 |
|  | 26-Hydroxycholesterol | 51.376 | 0.1987 |

**Chemical fingerprint of n-hexane fraction of NORE corresponding to Figure 6D**

| **Sl No.** | **Name of compounds** | **Retention time (min)** | **Relative abundance (%)** |
| --- | --- | --- | --- |
|  | 1-Methoxy-4,4-dimethyl-cyclohex-1-ene | 11.036 | 0.5675 |
|  | 2-Hexenoic acid, methyl ester, (E) | 12.399 | 0.2152 |
|  | Cyclohexane, 1-methyl-4-(1-methylethyl)-, trans- | 12.599 | 0.2799 |
|  | Ethanone, 1-(1-methylcyclohexyl)- | 13.575 | 0.0705 |
|  | 2-Pentene, 3-methyl-, (Z)- | 13.982 | 0.0808 |
|  | Propanoic acid, 2-methyl-, 2-ethylhexyl ester | 20.838 | 0.0608 |
|  | Tetradecane | 21.088 | 0.0196 |
|  | Nonanoic acid, 9-oxo-, methyl este | 22.114 | 0.0465 |
|  | Apocynin | 23.815 | 0.0229 |
|  | Benzoic acid, 4-hydroxy-3-methoxy-,methyl ester | 23.884 | 0.0461 |
|  | Fumaric Acid | 24.253 | 0.0268 |
|  | Cyclohexanol, 5-methyl-2-(1-methylethenyl)- | 25.761 | 0.0484 |
|  | Hexadecane | 25.973 | 0.0292 |
|  | Z-2-Dodecenol | 26.336 | 0.0716 |
|  | Dodecanoic acid | 26.636 | 0.0162 |
|  | Methyl tetradecanoate | 28.801 | 0.0311 |
|  | cis-9-Hexadecenoic acid | 30.346 | 0.0266 |
|  | Pentadecanoic acid, methyl ester | 30.940 | 0.0776 |
|  | 7-Octen-2-ol, 2,6-dimethyl- | 31.209 | 1.8326 |
|  | Cyclododecyne | 32.210 | 0.1142 |
|  | cis-7,cis-11-Hexadecadien-1-yl acetate | 32.535 | 0.19 |
|  | Hexadecanoic acid, methyl ester | 33.067 | 2.3782 |
|  | Pyrene, hexadecahydro- | 33.323 | 0.2452 |
|  | 1-Methylcarbazole | 33.742 | 0.3389 |
|  | n-Hexadecanoic acid | 34.687 | 2.4113 |
|  | Octadecanoic acid | 34.906 | 1.8577 |
|  | 9,12-Octadecadienoic acid (Z,Z)-,methyl ester | 36.538 | 16.987 |
|  | 9-Octadecenoic acid (Z)-, methyl ester | 36.595 | 3.8032 |
|  | Methyl stearate | 37.064 | 3.8229 |
|  | Murrayafoline A | 37.289 | 7.9699 |
|  | 9,17-Octadecadienal, (Z)- | 37.458 | 0.6458 |
|  | 9,12-Octadecadienoic acid (Z,Z)- | 38.302 | 20.9919 |
|  | Methyl 9.cis.,11.trans.t,13.trans.-octadecatrienoate | 39.635 | 1.1623 |
|  | cis-11-Eicosenoic acid, methyl ester | 39.885 | 1.6539 |
|  | Methyl 18-methylnonadecanoate | 40.304 | 0.9711 |
|  | 13-Tetradece-11-yn-1-ol | 41.061 | 0.4308 |
|  | trans,trans-1,8-Dimethylspiro[4.5]decane | 41.211 | 0.9931 |
|  | 9-Octadecyne | 42.256 | 0.8823 |
|  | Oleic Acid | 43.107 | 0.2633 |
|  | Acetophenone oxime, 3,5-di(tert-butyl)-4-hydroxy- | 43.507 | 5.2765 |
|  | cis-13-Eicosenoic acid | 44.095 | 0.3228 |
|  | 2-Methyl-1H-phenanthro[3,4-d)imidazol-10-ol | 44.308 | 0.1888 |
|  | Z,Z-2,13-Octadecadien-1-ol | 44.527 | 0.1384 |
|  | Isopropyl linoleate | 44.958 | 0.2763 |
|  | Tricosanoic acid, methyl ester | 45.202 | 0.1003 |
|  | beta.-Amyrin | 46.778 | 0.2082 |
|  | dl-.alpha.-Tocopherol | 48.880 | 1.1847 |
|  | Squalene | 50.025 | 0.4207 |
|  | Ibogaine | 50.231 | 0.2099 |
|  | alpha.-Amyrin | 50.869 | 1.3616 |
|  | Lupeol | 51.389 | 0.1166 |
