# Supplementary material for: Haloalkane induced hepatic insult in murine model: amelioration by Oleander through antioxidant and anti-inflammatory activities, an in vitro and in vivo study
Source: BMC Complement Altern Med. 2016 Aug 11;16:280. doi: 10.1186/s12906-016-1260-4 (PMC4982413; doi:10.1186/s12906-016-1260-4)
Supplement: Additional file 5: — Bioactivities of identified compounds. ▲ means increase; ▼ means decrease; ♦ means no change. (DOCX 33 kb) [file 12906_2016_1260_MOESM5_ESM.docx]

**Additional file 5**

Bioactivities of identified compounds. ▲ means increase; ▼ means decrease; ♦ means no change.

| **Compounds** | **Bioactivities** | **References** |
| --- | --- | --- |
| Apocynin | NF-κβ ▼, AP-1 ▼, TNF-α ▼, IL-1β ▼, IL-6 ▼, macrophages ▼, eosinophils ▼, IL-4 ▼, IL-5 ▼, IL-12 ▼, IL-13 ▼. | Kim, *et al*., 2012 |
|  | Antioxidant and free radical scavenger | Heumüller, *et al*., 2008 |
|  | ALT ▼, AST ▼, ALP ▼, liver inflammation and oxidative stress ▼ | Lu, *et al*., 2007 |
|  | Glomerular function ▲, TNF-α ▼, IL-1β ▼ |  |
| **Vaccenic acid** | Triglyceride ▼, IL-10 ▼ | Wang, *et al*., 2008 |
|  | IL-2 and TNF-α producing Th cell ▼ | Jaudszus, *et al*., 2012 |
| **Oleic acid** | TNF-α ▼, insulin ▼, glucose ▼ | Vassiliou, *et al*., 2009 |
|  | VCAM-1 ▼, E-selectin ▼, ICAM-1 ▼, neutrophil aggregation ▼, cell attachment ▼, phagocytic activity ▲, IL-2 ▼, IFN-γ ▼, T-cell proliferation ▼ | Carrillo, *et al*., 2012 |
|  | LDL ▼, phospholipase A(2) ▼, paraoxonase activity ▲, glycation of apolipoproteins ▼, LDL uptake ▼, MDA ▼, lipid hydroperoxides ▼ | Cho, *et al*., 2010 |
| **Tocopherol** | Glucose ▼, HbA1c ▼, insulin ▼, HOMA-IR ▼, cholesterol ▼, triglyceride ▼, catalase ▼, SOD ▲, GST ▲, lipid peroxidation ▼.  HYBRID and FRED docking studies: association of tocopherol with protein-tyrosine phosphatase-1B, peroxisome proliferator-activated receptor-γ and dipeptidyl peptidase IV. | Bharti, *et al*., 2013 |
|  | ALT ▼, argininosuccinic acid lyase ▼, histopathological signs of portal inflammation ▼, fatty changes ▼, necrosis ▼ | Tayal, *et al*., 2007 |
|  | Modulation ▲▼ of lymphocyte proliferation, IL-1, IL-2, PGE_2_ ▼, lipid peroxide ▼ | Meydani, *et al*., (1990) |
| **Vanillin** | ALT ▼, AST ▼, lipid peroxidation ▼, SOD ▲, catalase ▲, glutathione ▲, TNF-α ▼, IL-1β ▼, IL-6 ▼ | Makni, *et al*., 2011 |
|  | Glucose ▼, cholesterol ▼, HDL cholesterol ♦ | Duraipandi & Selvakumar (2012) |
| **Tetradecanoic acid (myristic acid)** | Free radical scavenging ▲, COX ▼ | Henry, *et al*., (2002) |
|  | Scavenger receptor BI ▼, cholesteryl ester ▼, sterol 27 hydroxylase ▲, 3-hydroxy-3-methyl glutaryl coenzyme A reductase ♦ | Loison, *et al*., 2002 |
|  | Modulation ▼▲ of IL-1, TNF-α, IL-8, p38, JNK kinases | Haversen, *et al*., 2009 |
| **Methylparaben** | Cytotoxicity ▼, ROS ▼, intranigral brain damage ▼, lipid peroxidation ▼, | Kopalli, *et al*., 2013 |
| **Murrayafoline** | IL-6 ▼, IL-12 ▼, p40 ▼, TNF-α ▼ | Thuy, *et al*., 2013 |
| **26-Hydroxycholesterol** | Cholesterol synthesis ▼, DNA synthesis ▼ | Javitt, *et al*., 1990 |
| **Isoeugenol** | NO ▼, iNOS ▼, NF-κβ activation ▼, p38 MAP kinase ▼ | Choi, *et al*., 2007 |
|  | CAT ▲, SOD ▲, GPX ▲, GR ▲, lipid peroxidation ▼ | Rauscher, *et al*., 2001 |
|  | AST ▼, ALP ▼, ALT ▼, GGT ▼, Bilirubin ▼, Lipid peroxidation ▼, Cholesterol ▼, SOD ▼, CAT ▼, GST ▼, GPx ▼ | Anbu and Anuradha, 2012 |
|  | TNF-α ▼ | Xiong, *et al*., (2000) |
|  | Acetylcholine-induced relaxations ▼, L-NAME-induced contractions ▼, acetylcholine ▲, glucose ♦, eNOS ▼, iNOS ▲, p22(phox) and gp91(phox) subunits of NADPH oxidase ▲ | Olukman, *et al*., 2010 |
| **Linoleic acid + tocopherol** | MDA ▲, Apolipoprotein B ▲ | Shadman, *et al*., (2013) |
| **Tryptamine** | NO ▼, PGE_2_ ▼, TNF-α ▼, IL-1β ▼, iNOS ▼, COX-2 ▼ | Vo, *et al*., 2014 |
| **Palmitic acid (hexadecanoic acid)** | phospholipase A2 ▼ (enzyme kinetic study) | Aparna, *et al*., (2012) |
| **Fumaric acid** | IL-4 ▲, IL-5 ▲, IL-10 ▲, IL-2 ♦, IFN-γ ♦ | Heiligenhaus, *et al*., (2005) |
|  | Macrophage inflammation ▼, IL-10 ▲ | Schilling, *et al*., (2006) |
|  | IL-6 ▼, TNF-α ▼, Lipid peroxidation ▼, | Šilhavý, *et al*., 2014 |
| **Amyrin** | ALP ▼, ACP ▼, Bilirubin ▼, SOD ▼, CAT ▼, GPx ▼, GR ▼, Lipid peroxidation ▼ | Singh, *et al*., 2015 |
|  | ALT ▼, AST ▼, GSH ▲ | Oliveira, *et al*., 2005 |
|  | Blood glucose load ▼, oral glucose tolerance ▲, entry of glucose from the intestine ▼ | Nair, *et al*., 2014 |
|  | IL-10 ▲, IL-1β ▼, endothelial growth factor ▼, COX-2 ▼, NF-κβ ▼ | Vitor, *et al*., 2009 |
| **Squalene** | DPPH ▼, peroxyl radical ▼, TEAC ▲, OH^●^ ▼ | Amarowicz (2009) |
|  | AST ▼, ALT ▼, ALP ▼, GGT ▼, Creatinine ▼, Urea ▼, Bilirubin ▼ | Sivakrishnan and Muthu, 2014 |
| **Lupeol** | HbA1c ▼, glucose ▼, NO ▼, insulin ▲, antioxidant enzymes ▲ | Gupta, *et al*., 2012 |
|  | ALT ▲, TNF-α ▲, IL-6 ▲,TLR-4 ▲, myeloid differentiation primary response gene 88 ▲, TIR-domain-containing adapter-inducing interferon-β ▲, IL-1 receptor-associated kinase ▲, TNF receptor associated factor 6 protein ▲ | Kim, *et al*., (2014) |
|  | PGE_2_ ▼, phagocytosis ▼, IL-2 ▼, IFN-γ, IL-4 ▼, IL-5 ▼, IL-13 ▼, NF-κβ translocation ▼ | Saleem (2009) |

**NF-κβ**= Nuclear factor kappa beta; **AP-1**= Activator protein-1 (transcription factor); **TNF-α**= Tumor necrosis factor alpha; **IL**= Interleukin; **ALT**= Alanine transaminase; **AST**= Aspartate transaminase; **ALP**= Alkaline phosphatase; **VCAM-1**= Vascular cell adhesion protein 1 (cell adhesion molecule); **ICAM-1**= Intercellular Adhesion Molecule 1; **IFN-γ**= Interferon gamma; **LDL**= Low density lipoprotein; **MDA**= Malondialdehyde; **HbA1c**= Glycated haemoglobin; **HOMA-IR**= Homeostatic model assessment and insulin resistance; **SOD**= Superoxide dismutase; **GST**= Glutathione S-transferase; **HDL**= High density lipoprotein; **COX**= Cyclooxygenase; **JNK**= c-Jun N-terminal kinase; **NO**= Nitric oxide; **ROS**= Reactive oxygen species; **iNOS**= Inducible nitric oxide synthase; **PGE_2_**= Prostaglandin E_2_; **CAT**= Catalase; **GPX**= Glutathione peroxidase; **GR**= Reduced glutathione; **GGT**= Gamma glutamyl transferase; **eNOS**= Endothelial nitric oxide synthase; **L-NAME**= Nω-Nitro-L-arginine methyl ester hydrochloride; **ACP**= Acid phosphatase; **TEAC**= Trolox equivalent antioxidant capacity; **OH**^●^= Hydroxyl radical; **TLR**= Toll like receptor.

# References

Ahmad A, Mondello S, Di Paola R, Mazzon E, Esposito E, Catania MA, Italiano D, Mondello P, Aloisi C, Cuzzocrea S (2012) Protective effect of apocynin, a NADPH-oxidase inhibitor, against contrast-induced nephropathy in the diabetic rats: a comparison with n-acetylcysteine. *Eur J Pharmacol*. 674, 397-406.

Amarowicz A (2009) Squalene: a natural antioxidant? *Eur J Lipid Sci Tech*. 111, 411–412.

Anbu S, Anuradha CV (2012) Protective effect of eugenol against alcohol-induced biochemical changes in rats. *Int J Res Biotech Biochem.* 2, 13-18.

Aparna V, Dileep KV, Mandal PK, Karthe P, Sadasivan C, Haridas M (2012) Anti-inflammatory property of n-hexadecanoic acid: structural evidence and kinetic assessment. *Chem Biol Drug Des.* 80, 434-9.

Bharti SK, Kumar A, Sharma NK, Prakash O, Jaiswal SK, Krishnan S, Gupta AK, Kumar A (2013) Tocopherol from seeds of *Cucurbita pepo* against diabetes: validation by *in vivo* experiments supported by computational docking. *J Formos Med Assoc*. 112, 676-690.

Carrillo C, Cavia Mdel M, Alonso-Torre S (2012) Role of oleic acid in immune system; mechanism of action; a review. *Nutr Hosp*. 27, 978-90.

Cho KH, Hong JH, Lee KT (2010) Monoacylglycerol (MAG)-oleic acid has stronger antioxidant, anti-atherosclerotic, and protein glycation inhibitory activities than MAG-palmitic acid. *J Med Food*. 13, 99-107.

Choi CY, Park KR, Lee JH, Jeon YJ, Liu KH, Oh S, Kim DE, Yea SS (2007) Isoeugenol suppression of inducible nitric oxide synthase expression is mediated by down-regulation of NF-kappaB, ERK1/2, and p38 kinase. *Eur J Pharmacol*. 576, 151-9.

Duraipandi S, Selvakumar V (2012) Design, synthesis and evaluation of vanillin Semicarbazones as hypoglycemic agents in alloxan-induced diabetes in rats. *J Pharma Res*. 5, 2441-2442.

Gupta R, Sharma AK, Sharma MC, Dobhal MP, Gupta RS (2012) Evaluation of antidiabetic and antioxidant potential of lupeol in experimental hyperglycaemia. *Nat Prod Res*. 26, 1125-9.

Haversen L, Danielsson KN, Fogelstrand L, Wiklund O (2009) Induction of proinflammatory cytokines by long-chain saturated fatty acids in human macrophages. *Atherosclerosis*. 202, 382-393.

Heiligenhaus A, Li H, Schmitz A, Wasmuth S, Bauer D (2005) Improvement of herpetic stromal keratitis with fumaric acid derivate is associated with systemic induction of T helper 2 cytokines. *Clin Exp Immunol*. 142, 180-7.

Henry GE, Momin RA, Nair MG, Dewitt DL (2002) Antioxidant and cyclooxygenase activities of fatty acids found in food. *J Agric Food Chem*. 50, 2231-2234.

Heumüller S, Wind S, Barbosa-Sicard E, Schmidt HH, Busse R, Schröder K, Brandes RP (2008) Apocynin is not an inhibitor of vascular NADPH oxidases but an antioxidant. *Hypertension*. 51, 211-7.

Jaudszus A, Jahreis G, Schlormann W, Fischer J, Kramer R, Degen C, Rohrer C, Roth A, Gabriel H, Barz D, Gruen M (2012) Vaccenic acid-mediated reduction in cytokine production is independent of c9,t11-CLA in human peripheral blood mononuclear cells. *Biochima et Biophysica Acta*. 1821, 1316-22.

Javitt NB (1990) 26-Hydroxycholesterol: synthesis, metabolism, and biologic activities. *J Lipid Res*. 31, 1527-33.

Kim SJ, Cho HI, Kim SJ, Kim JS, Kwak JH, Lee DU, Lee SK, Lee SM (2014) Protective effects of lupeol against D-galactosamine and lipopolysaccharide-induced fulminant hepatic failure in mice. *J Nat Prod*. 77, 2383-2388.

Kim SY, Moon KA, Jo HY, Jeong S, Seon SH, Jung E, Cho YS, Chun E, Lee KY (2012) Anti-inflammatory effects of apocynin, an inhibitor of NADPH oxidase, in airway inflammation. *Immunol Cell Biol*. 90, 441-448.

Kopalli SR, Noh SJ, Koppula S, Suh YH (2013) Methylparaben protects 6-hydroxydopamine-induced neurotoxicity in SH-SY5Y cells and improved behavioral impairments in mouse model of Parkinson's disease. *Neurotoxicology*. 34: 25-32.

Loison C, Mendy F, Sérougne C, Lutton C (2002) Dietary myristic acid modifies the HDL-cholesterol concentration and liver scavenger receptor BI expression in the hamster. *Br J Nutr*. 87, 199-210.

Lu LS, Wu CC, Hung LM, Chiang MT, Lin CT, Lin CW, Su MJ (2007) Apocynin alleviated hepatic oxidative burden and reduced liver injury in hypercholesterolaemia. *Liver Int*. 27, 529-37.

Makni M, Chtourou Y, Fetoui H, Garoui el M, Boudawara T, Zeghal N (2011) Evaluation of the antioxidant, anti-inflammatory and hepatoprotective properties of vanillin in carbon tetrachloride-treated rats. *Eur J Pharmacol*. 668, 133-9.

Meydani SN, Barklund MP, Liu S, Meydani M, Miller RA, Cannon JG, Morrow FD, Rocklin R, Blumberg JB (1990) Vitamin E supplementation enhances cell-mediated immunity in healthy elderly subjects. *Am J Clin Nutr*. 52, 557-63.

Nair SA, Sabulal B, Radhika J, Arunkumar R, Subramoniam A (2014) Promising anti-diabetes mellitus activity in rats of β-amyrin palmitate isolated from *Hemidesmus indicus* roots. *Eur J Pharmacol*. 734, 77-82.

Oliveira FA, Chaves MH, Almeida FR, Lima RC Jr, Silva RM, Maia JL, Brito GA, Santos FA, Rao VS (2005) Protective effect of alpha- and beta-amyrin, a triterpene mixture from *Protium heptaphyllum* (Aubl.) March. trunk wood resin, against acetaminophen-induced liver injury in mice. *J Ethnopharmacol*. 98, 103-8.

Olukman M, Orhan CE, Çelenk FG, Ulker S (2010) Apocynin restores endothelial dysfunction in streptozotocin diabetic rats through regulation of nitric oxide synthase and NADPH oxidase expressions. *J Diabetes Complications*. 24, 415-423.

Rauscher FM, Sanders RA, Watkins JB (2001) Effects of isoeugenol on oxidative stress pathways in normal and streptozotocin-induced diabetic rats. *J Biochem Mol Toxic*. 15, 159-164.

Saleem M (2009) Lupeol, a novel anti-inflammatory and anti-cancer dietary triterpene. *Cancer Lett*. 285, 109-115.

Schilling S, Goelz S, Linker R, Luehder F, Gold R (2006) Fumaric acid esters are effective in chronic experimental autoimmune encephalomyelitis and suppress macrophage infiltration. *Clin Exp Immunol*. 145, 101-107.

Shadman Z, Taleban FA, Saadat N, Hedayati M (2013) Effect of conjugated linoleic acid and vitamin E on glycemic control, body composition, and inflammatory markers in overweight type2 diabetics. *J Diabetes Metab Disord*. 12, 42.

Šilhavý J, Zídek V, Mlejnek P, Landa V, Šimáková M, Strnad H, Oliyarnyk O, Škop V, Kazdová L, Kurtz T, Pravenec M (2014) Fumaric acid esters can block pro-inflammatory actions of human CRP and ameliorate metabolic disturbances in transgenic spontaneously hypertensive rats. *PLoS One*. 9, e101906.

Singh D, Arya PV, Sharma A, Dobhal MP, Gupta RS (2015) Modulatory potential of α-amyrin against hepatic oxidative stress through antioxidant status in Wistar albino rats. *J Ethnopharmacol*. 161, 186-93.

Sivakrishnan S, Muthu AK (2014) Evaluation of hepatoprotective activity of squalene isolated from *Albizia procera* against paracetamol induced hepatotoxicity on wistar rats. *World J Pharmacy Pharma Sci*. 3, 1351-1362.

Tayal V, Kalra BS, Agarwal S, Khurana N, Gupta U (2007) Hepatoprotective effect of tocopherol against isoniazid and rifampicin induced hepatotoxicity in albino rabbits. *Indian J Exp Biol*. 45, 1031-1036.

Thuy TT, Cuong NM, Toan TQ, Thang NN, Tai BH, Nhiem NX, Hong HJ, Kim S, Legoupy S, Koh YS, Kim YH (2013) Synthesis of novel derivatives of murrayafoline A and their inhibitory effect on LPS-stimulated production of pro-inflammatory cytokines in bone marrow-derived dendritic cells. *Arch Pharm Res*. 36, 832-9.

Vassiliou EK, Gonzalez A, Garcia C, Tadros JH, Chakraborty G, Toney JH (2009) Oleic acid and peanut oil high in oleic acid reverse the inhibitory effect of insulin production of the inflammatory cytokine TNF- α both *in vitro* and *in vivo* systems. *Lipids Health Dis*. 8, 25.

Vitor CE, Figueiredo CP, Hara DB, Bento AF, Mazzuco TL, Calixto JB (2009) Therapeutic action and underlying mechanisms of a combination of two pentacyclic triterpenes, α- and β-amyrin, in a mouse model of colitis. *Br J Pharmacol*. 157, 1034-1044.

Vo VA, Lee JW, Park JH, Kwon JH, Lee HJ, Kim SO, Kwon YS, Chun W (2014) N-(p-Coumaryol)-Tryptamine suppresses the activation of JNK/c-Jun signaling pathway in LPS-challenged RAW264.7 cells. *Biomol Ther*. 22, 200-206.

Wang Y, Lu J, Ruth MR, Goruk SD, Reaney MJ, Glimm DR, Vine DF, Field CJ, Proctor SD (2008) Trans-11 vaccenic acid dietary supplementation induces hypolipidemic effects in JCR:LA-cp rats. *J Nutrition*. 138, 2117-2122.

Xiong Q, Fan W, Tezuka Y, Adnyana IK, Stampoulis P, Hattori M, Namba T, Kadota S (2000) Hepatoprotective effect of *Apocynum venetum* and its active constituents. *Planta Medica*. 66, 127-133.
